# Efficacy and safety of hydroxychloroquine as add-on therapy in uncontrolled type 2 diabetes patients who were using two oral antidiabetic drugs

**DOI:** 10.1007/s40618-020-01330-5

**Published:** 2020-06-27

**Authors:** H. N. Chakravarti, A. Nag

**Affiliations:** Department of Medicine, Medical College and Hospital, Kolkata, West Bengal India

**Keywords:** Hydroxychloroquine, Type 2 diabetes, HbA1c, FPG, PPG

## Abstract

**Objective:**

To evaluate the Safety and Efficacy of Hydroxychloroquine as add-on therapy in uncontrolled type 2 diabetes patients who were using two oral antidiabetic drugs.

**Materials and methods:**

This was a double blind, placebo controlled, parallel group study in 304 inadequately controlled type 2 diabetes (T2DM) subjects with two oral antidiabetic drugs (glimepiride 4 mg and metformin 500 mg) were randomised to hydroxychloroquine (HCQ) 200 mg, 300 mg, 400 mg once daily (OD) or placebo. Dose of hydroxychloroquine was selected as per body weight of the subject. Primary end point was glycated haemoglobin (HbA1c) change at week 12 from baseline. Secondary endpoint was change in fasting plasma glucose (FPG), post prandial plasma glucose (PPG), body weight and any adverse reaction including no of hypoglycemic events, as well as a change in the percentage of subjects with A1C < 7.0% and > 6.5% after 12 weeks of treatment.. In follow-up of 400 mg once daily was once again divided to 200 mg twice daily (BD) to study the effect on tolerability profile for further 12 weeks.

**Results:**

Hydroxychloroquine was associated with significant reduction in HbA1c from baseline (7–8.5%) in 12 weeks −0.78%, −0.91% and 1.2% for hydroxychloroquine 200 mg, 300 mg and 400 mg OD, respectively, versus 0.13% with placebo (*P* < 0.005). FPG and PPG were reduced by −25 to −38 mg/dl and 34–53 mg/dl, respectively. Body weight also reduced in each group of HCQ. Hypoglycemia was reported only with 300 mg (1.2%) and 400 mg (2.1%) group of HCQ. It was observed that patients who complains with mild GI disturbance with HCQ 400 mg glycemic efficacy was maintained with 200 mg BD with significant relief of the symptoms.

**Conclusion:**

Hydroxychloroquine added to sulphonylurea and metformin, improves glycemic control significantly in T2DM patients. Glycemic effect of different dose of hydroxychloroquine is dose dependent. The safety/tolerability profile of hydroxychloroquine was favourable except GI disturbance which is more frequent with 400 mg. This can be avoided with 200 mg BD without compromise on efficacy.

## Introduction

The International Diabetes Federation (IDF) reported that, at present, there are 415 million people worldwide suffering from DM, aged between 20 and 79 years old, the global prevalence being of 8.8%, and it is estimated that in 2040 their number will grow up to 642 million, with a prevalence of 10.4% [1]. In India as per 2015 scenario, 69.1 million cases of diabetes were reported [2].

Metformin is known for the first-line therapy for type 2 diabetes mellitus (non-insulin dependent diabetes mellitus). It is the common drug prescribed worldwide. Metformin is a biguanide agent, and it lowers both basal and post-prandial plasma glucose (PPG) [3]. American Diabetes Association (ADA) recommends metformin to treat individuals diagnosed with type 2 diabetes and recommends that glycated haemoglobin (HbA1c) should be maintained below or around 7% [4].

The latest position statement from the ADA recommends initiating a combination of two non-insulin agents when the patients have a high baseline HbA1c (≥ 9.0%) because these patients are unlikely to achieve target A1c with metformin monotherapy [5]. It has been hypothesized that combining metformin and an agent from another class with a different mechanism of action may help to preserve β-cell function and thereby maintain a long-term glycaemic efficacy or ‘durability’ [6]. The major classes of oral antidiabetic medications include biguanides, sulfonylureas, meglitinide, thiazolidinedione (TZD), dipeptidyl peptidase 4 (DPP-4) inhibitors, sodium-glucose cotransporter (SGLT-2) inhibitors, and α-glucosidase inhibitors. If the HbA1C level rises to 7.5% while on medication or if the initial HbA1C is ≥ 9%, combination therapy with two oral agents, or with insulin, may be considered [7].Even before initiating insulin therapy, GLP-1 receptor agonists are recommended as add-on therapy for patients who do not achieve their A1C target after 3 months of metformin therapy [8].

However, most patients initially respond to sulfonylureas and/or metformin at the starting stage, later on these agents lose their effectiveness with time; comparable long-term data is not yet available for the alpha glucosidase inhibitors, the meglitinides and the thiazolidinediones [9]. Insulin therapy is not only costly but is not preferred due to poor patients’ compliance in parenteral application. New generation thiazolidinediones class of antidiabetic drugs, though useful in glycemic control, is associated with several adverse effects such as excessive risk of congestive heart failure, acute myocardial infarction, increased rate of bone loss and liver toxicity [9]. In the light of failure of monotherapy of antidiabetic drugs in glycemic control and increased adverse effects when administered at high doses of antidiabetic drugs for getting better glycemic control, newer medications for diabetes are needed, which will have good anti-hyperglycemic effect, as well as good tolerability profile. Though there is already an option of GLP-1 RA and SGLT-2 inhibitors and apart of the efficacy of glycemic control they also offer proven cardiovascular and renal benefits, but due to their exorbitant cost they are beyond the reach of many patients especially in developing country like India. Therefore the need of adding second oral antihyperglycemic agent is required when metformin or sulfonylureas do not achieved a HbA1C target or metformin monotherapy at maximal tolerated dose over 3–6 months.

In 2014 hydroxychloroquine was approved by Drug Controller General of India (DCGI) for the management of Type 2 Diabetes (T2DM) as an adjacent to diet and exercise to improve glycemic control in patients with T2DM on combination of sulfonylurea and metformin and even endorsed by RSSDI (Research Society for the Study of Diabetes in India) clinical practice recommendations for the management of type 2 diabetes mellitus 2017 [10]. Hydroxychloroquine (HCQ) improve glucose tolerance and insulin sensitivity by inhibition of insulin degradation. It slows breakdown of the internalized insulin-receptor complex and has a modest effect on reducing glycemic parameters along with reduction of pro-inflammatory markers [11]. An Indian randomized controlled phase 3 trials showed that HCQ (400 mg) as compared to pioglitazone (15 mg) lowers HbA1c, LDL cholesterol levels and lead to weight loss in patients with type 2 diabetes [12].

Objective of the current study was to evaluate the Safety and Efficacy of Hydroxychloroquine as add-on therapy in uncontrolled type 2 diabetes patients who were using two oral antidiabetic drugs. A different dose was used to determine the dose dependent efficacy and safety which has compared with placebo.

## Material and methods

This was a randomized, double blind, placebo controlled, parallel group, single centre, prospective study. Subjects were randomised to three groups of hydroxychloroquine depending on dose of 200 mg, 300 mg and 400 mg, along with placebo group. In follow-up of 400 mg once daily was once again divided to 200 mg twice daily (BD) to study the effect on tolerability profile for further 12 weeks.

The study was initiated only after obtaining necessary approvals from Institutional ethics committee of Medical College, Kolkata (Ref No. MC/kol/IEC/Non-spon/641/11-2017). Patient information sheet (PIS) was discussed with each enrolled patients or legally acceptable representative (LAR)/impartial witness (in case of illiterate patients) as applicable. The complete informed consent process was in adherence with the current regulatory requirement (s). The study was conducted in accordance with the principles in the Declaration of Helsinki and was consistent with good clinical practices and applicable regulatory requirements.

At screening visit, demographic characteristics, medical history, and details of previous and current medication (s) including details of metformin and sulfonylurea dose and duration was recorded. Complete physical examination was performed; vital signs, body weight, was recorded. Pathological test was performed for HbA1c, FPG, 2-h PPG (post-prandial glycemia After a standardized meal) at different interval and requirements of the study from Kolkata Medical College pathology department or NABL (National Accreditation Board for Testing and Calibration Laboratories) accredited pathological laboratory as per the convenience and economical affordability of subjects. Electrocardiogram (ECG) was recorded at this visit. Detailed ophthalmological examination (visual acuity test, fundoscopic test, visual field test, expert slit lamp test and amsler grid test) was performed to identify any grade of diabetic retinopathy and maculopathy. All female patients of child bearing potential was undergo a urine pregnancy test (UPT). A negative urine pregnancy test was obtained before including any patient on the study. Compliance to study medications was assessed based on returned used study drugs and patients diary.

### Dose selection criteria

The most recent 2011 guidelines from the American Academy of Ophthalmology (AAO) recommend the dose of HCQ ≤ 6.5 mg/kg based on ideal body weight to minimize toxicity [13]. Even 2016 guideline recommend HCQ ≤ 5 mg/kg of base ingredient on real body weight [14]. Based on this for initiating treatment patients who had body weight ≥ 60 kg was selected for HCQ 400 mg and 300 mg, patients < 60 kg body weight was selected suitable for 300 mg and 200 mg only.

### Study population

Eligible patients were aged 18–75 years with T2DM, who were inadequately controlled (HbA1c ≥ 7–8.5%) on stable combination therapy with glimepiride 4 mg and metformin 500 mg. This two therapy dosage was chosen as this are two most frequently prescribed and tolerated dose specially in India. Patients were excluded if any history or presence of any retinopathy of any grade including diabetic retinopathy requiring laser therapy, evidence of an imminent need for retinal laser therapy, uncorrected visual acuity < 20/100, abnormal visual fields, difficulty to examine optic disc, or evidence of retinal pigment epithelial abnormalities and patients with history or risk of macular oedema. Patients with recent (< 1 year) CV events i.e., myocardial infarction/ACS, stroke or has undergone coronary artery bypass surgery, percutaneous transluminal coronary angioplasty or transient ischemic attack, or history of congestive heart failure, or unstable angina, abnormal renal function (serum creatinine ≥ 1.5 mg/dl), history of symptomatic autonomic neuropathy or chronic gastroparesis, active gastrointestinal disorders (gastric and duodenal ulcer) were excluded from the study.

### Study end points

Primary end point was glycated haemoglobin (HbA1c) change at week 12 from baseline. Secondary endpoint was change in fasting plasma glucose (FPG), post prandial plasma glucose (PPG), body weight and any adverse reaction including no of hypoglycemic events, as well as a change in the percentage of subjects with A1C < 7.0% and > 6.5% after 12 weeks of treatment.

### Safety assessments

Safety assessments included the number of patients with adverse events (AEs), including AEs of special interest pre specified for inferential testing without multiplicity control (symptomatic hypoglycaemia (defined as episodes with clinical symptoms reported by the investigator as hypoglycaemia; biochemical documentation not required), and AEs associated with gastro intestinal disturbance. Investigator have confirmed hypoglycaemia whenever reported by patients (initially detected by SMBG) from a NABL accredited pathological laboratory as per the convenience and economical affordability of subjects.

### Statistical methods

Descriptive statistics was used to compare the demographic and baseline disease characteristics. Data was presented in terms of mean ± SD, median, percentiles or range for continuous variables and percentage for categorical variables. All the patients was compared at baseline for homogeneity using ANOVA test for continuous variables and Chi square test or Fisher’s exact test for categorical variable. For all statistical tests the significance level was set as 0.05. SAS 9.3 was used for statistical analysis.

### Sample size calculation

Sample size of 61 patients in each group was required to give more than 80% power with *α* = 0.017 (adjusted for three potential comparisons) to detect a treatment difference of 0.4% in HbA1c for Hydroxychloroquine 200 mg, Hydroxychloroquine 300 mg and Hydroxychloroquine 400 mg with placebo and assumed a standard deviation of 1%.

### Randomization procedure

Randomization codes was generated using block randomization programme in SAS 9.3^®^. Patient’s identification number were used in patient’s diary. All patients were received stable dose of metformin 500 mg and glimepiride 4 mg along with one of the therapies, either placebo, or active hydroxychloroquine 200 mg, or 300 mg or 400 mg tablets. The study drugs including placebo and active drug was arranged by the investigators at their own cost from local generic manufacturing agency.

## Results

**Fig. 1:**
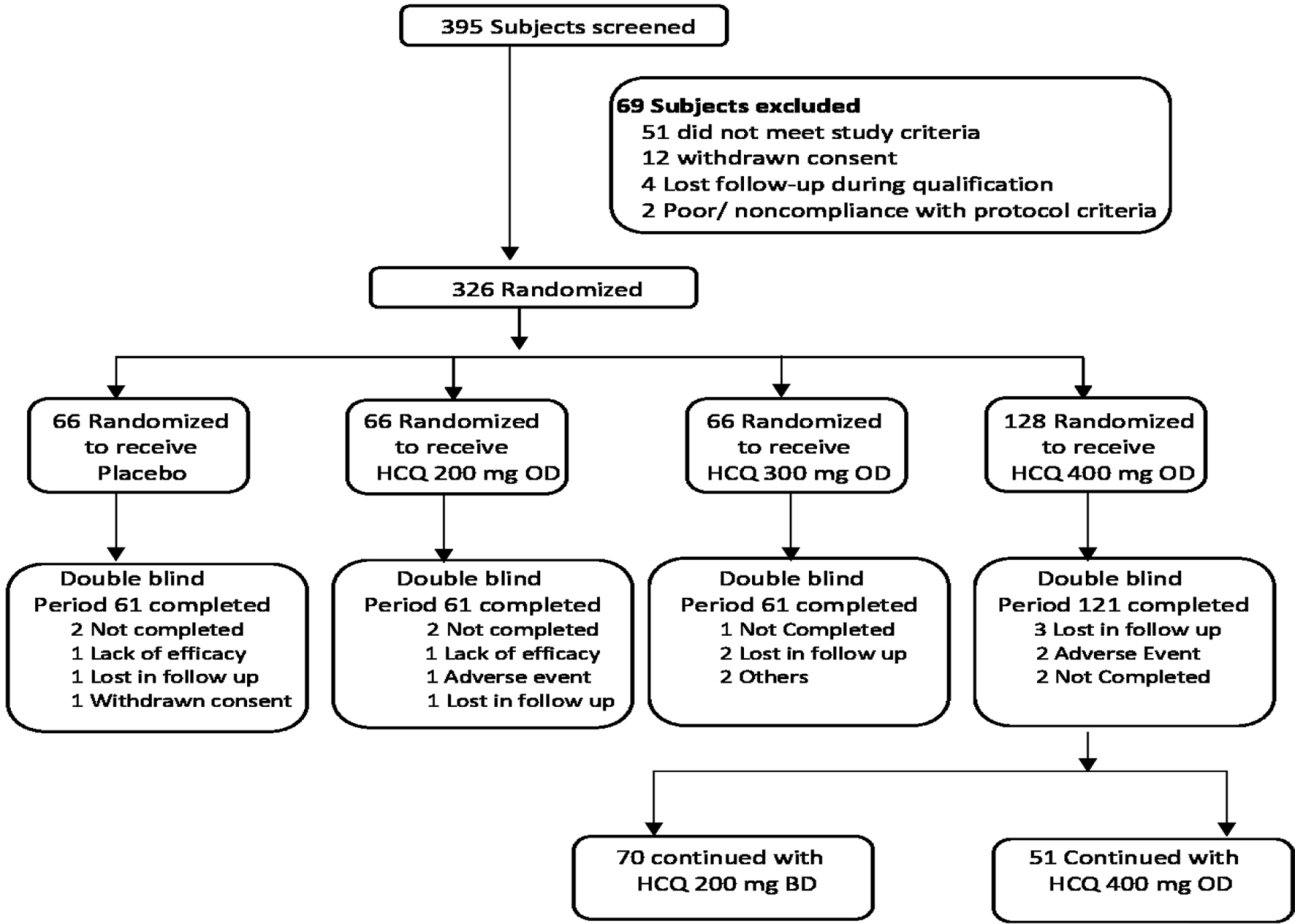
Patient disposition

Of 395 patients screened for the treatment cohort, 326 were randomly assigned (Fig. 1). Demographic and baseline characteristics are reported in (Table 1). Baseline demographics were generally similar between groups. Patient’s disposition was details in (Fig. 1).

**Table 1:** Demographic and baseline characteristics of randomly assigned patients in the treatment cohort

Characteristics	Met + SU + placebo (*N* = 61)	Met + SU + HCQ 200 mg (*N* = 61)	Met + SU + HCQ 300 mg (*N* = 61)	Met + SU + HCQ 400 mg (*N* = 121)	*P* value
Age (years)	53.36 ± 7.8	53.36 ± 8.5	51.76 ± 8.0	52.96 ± 9.6	0.953
Sex (male/female)	32/29	39/22	41/20	81/40	0.305
Weight (kg)	62.6 ± 6.1	59.6 ± 6.7	61.6 ± 5.4	70.6 ± 8.2	0.191
BMI (kg/m^2^)	25.1 ± 2.5	24.3 ± 2.1	24.8 ± 2.3	25.5 ± 2.6	0.219
Duration of type 2 diabetes (years)	6.46 ± 5.0	5.66 ± 5.0	6.16 ± 4.7	6.46 ± 5.7	0.329
HbA1C (%)	7.72 ± 0.5	7.83 ± 0.5	7.89 ± 0.5	7.93 ± 0.5	0.047
FPG (mg/dl)	157 ± 32	164 ± 38	168 ± 42	170 ± 45	0.030
PPG (mg/dl)	269 ± 58	275 ± 53	278 ± 56	282 ± 51	0.052
Hypertension	36 (59%)	48 (78%)	47 (77%)	96 (79%)	0.917
Dyslipidemia	38 (62%)	46 (75%)	44 (72%)	91 (75%)	0.879

Data are means ± SD, *n* (%), or median (interquartile range). Analysed by one-way ANOVA

*BMI* body mass index, *HbA1c* glycated haemoglobin, *FPG* fasting plasma glucose, *PPG* post-prandial glucose

Mean age of patients and gender distribution were almost similar in all groups. All group subjects had comparable FPG, PPG and HbA1c at the baseline. As per body weight hydroxychloroquine dose were selected. Patient’s ≥ 60 kg were selected for hydroxychloroquine 400 mg dose. Intergroup p value for demographic and baseline characteristic were statistically non-significant (Table 1).

**Fig. 2:**
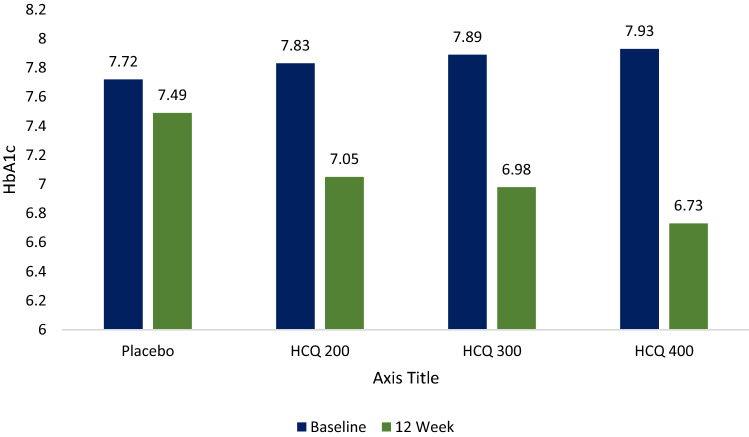
Change in HbA1c from baseline to week 12 in subjects with type 2 diabetes on metformin and sulfonylurea after addition of hydroxychloroquine or placebo

Hydroxychloroquine was associated with significant reduction in HbA1c from baseline (7–8.5%) in 12 weeks −0.78%, −0.91% and 1.2% for hydroxychloroquine 200 mg, 300 mg and 400 mg OD, respectively, versus 0.13% with placebo (*P* < 0.005). HbA1c reduction was highest with HCQ 400 mg as compare to other group. It has observed a dose dependent reduction across HCQ group and was statically significant as compared to placebo group (Table 2) (Fig. 2).

**Table 2:** Change in weight and glycemic parameters from baseline at Week 12

Characteristics	Met + SU + placebo (n = 61)			Met + SU + HCQ 200 mg (*N* = 61)			Met + SU + HCQ 300 mg (*N* = 61)			Met + SU + HCQ 400 mg (*N* = 121)			*P* value
	Baseline	12 week	*P* value	Baseline	12 week	*P* value	Baseline	12 week	*P* value	Baseline	12 week	*P* value	
Weight (kg)	62.6 ± 6.1	63 ± 5.9	0.246	59.6 ± 6.7	58.9 ± 6.5	0.712	61.5 ± 5.4	60.4 ± 5.1	0.241	70.7 ± 8.2	68.4 ± 7.6	0.213	0.113
HbA1C (%)	7.72 ± 0.5	7.49 ± 0.02	0.018	7.83 ± 0.5	7.05 ± 0.05	< 0.005	7.89 ± 0.5	6.98 ± 0.05	< 0.001	7.93 ± 0.5	6.73 ± 0.05	< 0.001	< 0.005
FPG (mg/dl)	157 ± 21	146 ± 16	0.014	164 ± 38	139 ± 29	< 0.005	168 ± 42	135 ± 31	< 0.001	170 ± 45	134 ± 34	< 0.001	< 0.005
PPG (mg/dl)	269 ± 36	257 ± 19	0.019	275 ± 53	241 ± 41	< 0.005	278 ± 56	234 ± 46	< 0.001	282 ± 51	229 ± 49	< 0.001	< 0.005

After further 12 weeks continuation glycemic parameters was almost similar in both HCQ 400 mg OD group and HCQ 200 mg BD group and *P* value of in-between group was non-significant (Table 3).

**Table 3:** Change in weight and glycemic parameters from baseline at further follow-up at Week 12

Characteristics	Met + SU + HCQ 200 mg BD (*N* = 70)			Met + SU + HCQ 400 mg OD (*N* = 51)			*P* value
	Baseline	12 week	*P* value	Baseline	12 week	*P* value	
Weight (kg)	70.7 ± 8.2	69.4 ± 7.5	0.010	70.7 ± 8.2	68.8 ± 7.1	0.12	0.219
HbA1C (%)	7.93 ± 0.5	7.31 ± 0.5	< 0.005	7.93 ± 0.5	7.39 ± 0.5	< 0.005	0.749
FPG (mg/dl)	170 ± 45	114 ± 21	< 0.005	170 ± 45	111 ± 26	< 0.005	0.843
PPG (mg/dl)	282 ± 51	189 ± 36	< 0.005	282 ± 51	182 ± 34	< 0.005	0.894

**Fig. 3:**
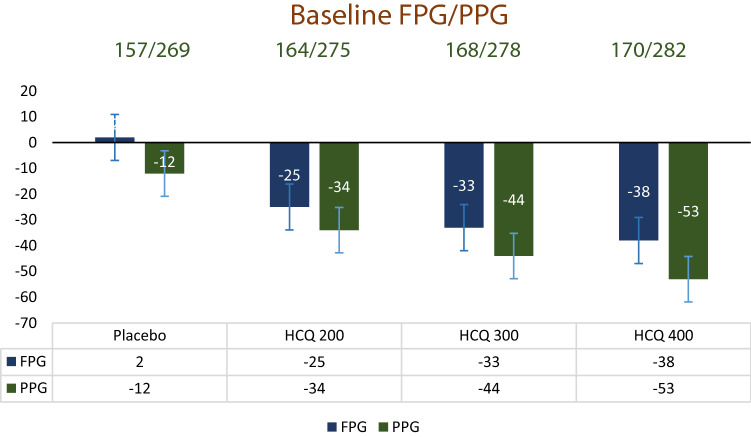
Mean change in FPG and PPG from baseline to week 12 in subjects with type 2 diabetes on metformin and sulfonylurea after addition of hydroxychloroquine or placebo

Significantly greater mean reductions in FPG and PPG were observed with all doses of hydroxychloroquine (−25 mg/dl to −38 mg/dl and −34 mg/dl to −53 mg/dl, respectively) compared with placebo (+ 2 mg/dl and −12 mg/dl respectively) (Fig. 3). FPG and PPG reduction were gradually increase with the increasing dose of hydroxychloroquine and the intergroup p value was statistically significant. Even after 12 week further follow-up of FPG and PPG were almost similar in both HCQ 400 mg OD group and HCQ 200 mg BD group and p value of in-between group was also non-significant (Table 3).

Efficacy was further assessed by determining the proportion of subjects who achieved an A1C < 7.0% at week 12 of the study (Table 4).

**Table 4:** Percentage of patients with HbA1c < 7%

Variables	Met + SU + placebo (*N* = 61)	Met + SU + HCQ 200 mg (*N* = 61)	Met + SU + HCQ 300 mg (*N* = 61)	Met + SU + HCQ 400 mg (*N* = 121)
HbA1C < 7.0%	0	4 (6.5%)	12 (19.6%)	32 (26.4%)

**Fig. 4:**
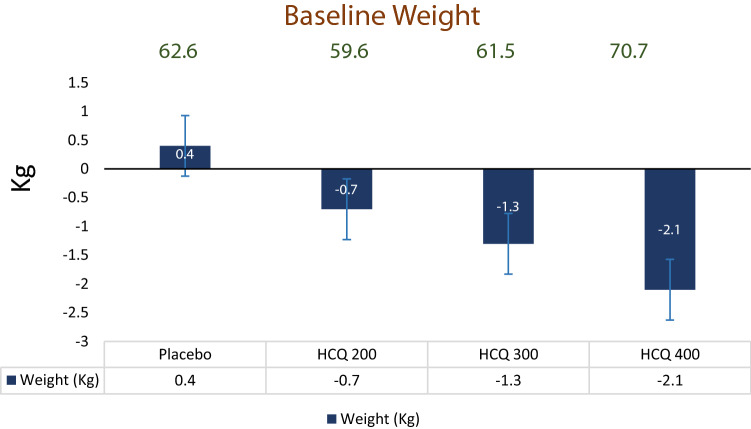
Change in body weight from baseline to week 12 in subjects with type 2 diabetes on metformin and sulfonylurea after addition of hydroxychloroquine or placebo

Body weight reductions were seen in all hydroxychloroquine groups relative to placebo (Fig. 4). Hydroxychloroquine reduced body weight from baseline; these reductions were −0.7 to −2.1 kg at week 12. Were as in placebo group 0.4 kg weight was increased from baseline. Weight loss appeared to be greatest in subjects in the hydroxychloroquine 400 mg groups. Even when hydroxychloroquine dose was further followed up for 12 weeks more there was not much significant difference was noticed in between hydroxychloroquine 200 mg BD group and hydroxychloroquine 400 mg OD group. All patients were on strict diet regimen which was monitored with periodic interval and was strictly advised for adequate exercise regime.

Incidence of AE was almost in similar pattern across the hydroxychloroquine group, except there was a higher gastrointestinal AE with higher dose of hydroxychloroquine (Table 5). Mild to moderate AE was determined by the investigators depending upon the severity and intensity of the event. Symptomatic hypoglycemia was considered as one of the most serious AE but no symptomatic hypoglycemia was requires additional medical assistance. It has been observed that there was no discontinuation of the drug because of any AE. Hydroxychloroquine was well tolerated in this study, with no evident increase in overall AE incidence compared with placebo, across the once-daily treatment groups.

**Table 5:** Summary of AEs after 12 weeks of treatment

	Met + SU + placebo (*N* = 61)	Met + SU + HCQ 200 mg (*N* = 61)	Met + SU + HCQ 300 mg (*N* = 61)	Met + SU + HCQ 400 mg (*N* = 121)
Subjects with an AE	21 (34.4%)	12 (19.6%)	17 (27.8%)	38 (31.4%)
Subjects with a moderate AE	0	2	3	5
Most common AEs				
Headache	2	1	0	1
Fatigue	1	1	0	0
Weight increased	12	1	0	0
Symptomatic hypoglycemia events	0	2	3	5
Severe hypoglycemia (that requires assistance from another person to treat)	0	0	0	0
Gastrointestinal (GI) disturbances	1	2	6	16
Flatulence	0	2	1	4
Constipation	0	0	1	3
Upper respiratory tract infection	1	1	0	1
Rise in CPK level	1	0	0	0
Urinary tract infection	2	0	1	1
Dyslipidemia	1	0	0	0
Deaths	0	0	0	0
Pain in extremity	1	0	0	0
Pigmentation	0	0	0	2
Non-proliferative diabetic retinopathy	1	0	0	0
Chest pain	1	0	0	0

Data are *n* (%) unless otherwise indicated

The main purpose of further 12 week study was to determine that what the glycemic control was if 400 mg OD further divided to 200 mg BD with a special interest to observe the effect on AE which was related to higher dose of hydroxychloroquine. It was been observed that with divided dose of hydroxychloroquine 200 mg BD the gastrointestinal side effects was drastically bring down (Table 6).

**Table 6:** Summary of AEs at further follow-up at week 12

	Met + SU + HCQ 200 mg BD (*N* = 70)	Met + SU + HCQ 400 mg OD (*N* = 51)
Symptomatic hypoglycemia events	2	3
Gastrointestinal (GI) disturbances	2	8
Flatulence	0	1
Constipation	0	1
Diarrhoea	0	1
Pigmentation	0	1

## Discussion

Diabetes is a huge and growing problem, and the cost to society is very high and escalating. Type 2 diabetes is a major risk factor for developing both microvascular and macrovascular complications (15). The primary goal of treatment is to target glycemic control by maintaining the HbA1c level near 6–7% to decrease the incidence of microvascular and macrovascular complications without predisposing patients to hypoglycemia (16). Subjects with T2DM often begins treatment by taking oral agents, usually metformin or a sulfonylurea, and then progress to the combination of these two agents.

Hydroxychloroquine was approved by Drug Controller General of India (DCGI) as an adjunct to diet and exercise to improve glycemic control of patients on metformin, sulfonylurea combination in T2DM. By increasing the intracellular pH, Hydroxychloroquine inhibits various insulin degrading enzymes [17] and thus inhibits insulin degradation [18]. The present study demonstrated the beneficial effect on overall glycemic control of additional hydroxychloroquine therapy in subjects insufficiently controlled by metformin and sulfonylurea combination. Even a significant percentage of patients had achieved target HbA1c < 7.0% at week 12 of the study.

Rise in inflammatory markers may occur in diabetes and even in prediabetes stage. This can be postulated from the face that both micro vascular and macro vascular complications are noted in prediabetes stage. Hydroxychloroquine lowers pancreatic levels of CRP, TNF alpha, PG, IL-1 and IL-6 [19, 20]. Hydroxychloroquine increases total concentration of circulating adiponectin levels by 18.7% [21]. It reduces adipocyte inflammation and islet inflammation which in turn reduces insulin resistance and insulin insufficiency and thus works in T2DM. In a study conducted by Rekedal et al. [22] in RA with diabetes, 0.66% reduction in baseline HbA1c post 12 months with hydroxychloroquine use was observed. Hydroxychloroquine shown favourable effects on both glucose control and lipid profiles beyond its anti-inflammatory role. Use of Hydroxychloroquine in T2DM is independently associated with a significant decrease in LDL, total cholesterol, LDL/HDL and total cholesterol/HDL [12]. It also improves insulin sensitivity in non-diabetic obese individual [23].

In multiple recently conducted observational studies, hydroxychloroquine at 400 mg dose exhibit a potent glycemic control, when compared to newer DCGI approved antidiabetic agents like teneligliptin [24, 25].

Efficacy of hydroxychloroquine was dose dependent. Higher the dose of hydroxychloroquine provides higher reduction of glycemic parameters. Even in another Indian trial hydroxychloroquine 400 mg reduced HbA1c at tune of 1.3% whereas hydroxychloroquine 200 mg reduced the same by o.8% over 24 week [26]. In this RCT trial also it has been seen that the glycemic effect of hydroxychloroquine was dose dependent. There was a significant HbA1c reduction with all the dose of hydroxychloroquine. With regard to the anti-inflammatory effect of hydroxychloroquine in T2DM, Amit Gupta [27] has recently shown that diabetic patients with higher baseline levels of high sensitivity C-reactive protein (hs-CRP > 3 mg/l) exhibited a more pronounced, although not significant, improvement in glucose control from baseline to 48 weeks after the initiation of hydroxychloroquine therapy, as compared to patients with lower baseline levels of hs-CRP (≤ 3 mg/l).

The current RCT trial is the first trial to show that even type 2 diabetes patients who were below 60 kg of body weight can get the benefit from lower dose of hydroxychloroquine i.e., hydroxychloroquine 200 mg and hydroxychloroquine 300 mg. We have shown that weight based dosing can be an ideal option for choosing hydroxychloroquine as an add-on antidiabetic treatment.

The half-life of Hydroxychloroquine is 50 days. In the setting of RA and lupus the drug is slow acting but in a diabetic patients Quatraro et al. [28] showed that Hydroxychloroquine decreased the glucose profile within 10–14 days. In the Indian study use of hydroxychloroquine as an add-on to metformin and sulfonylurea resulted in HbA1c reduction of 0.56% at week 12 and 0.87% at 24 weeks. In this trial a statistically significant reduction was observed even at week 12 among all across hydroxychloroquine group [12].

In the present study, across the hydroxychloroquine group body weight was decreased significantly. The reason that weight was found to be decreased by hydroxychloroquine treatment in the present study is not clear. A study published in 2015 in the journal Heart Rhythm found that the mice who consumed the Hydroxychloroquine do not show any weight gain or weight loss [29].

Although there was no incidence of ‘major hypoglycemia’, all episodes of hypoglycemia resolved or subsided after the patient’s ingested glucose or food at their own discretion. The incidence rates of hypoglycemia were low throughout the study and did not increase during any specific period. The probable reason for less incidence of hypoglycaemia with Hydroxychloroquine may because of its actions at peripheral levels i.e. insulin degradation in the target cells and thus facilitate insulin recycling, unlike any secretagogue. Mirza S et al. [30] mentioned odd ratios of IL-6 and other cytokines is a sensitive physiological markers of subclinical inflammation, associated with hyperglycemia, insulin resistance and overt T2DM. Effect of hydroxychloroquine on IL-6 and cytokine may responsible for less hypoglycemia as seen in several trails including the current one. In the Indian RCT study the incidence of hypoglycemia was 0% in the hydroxychloroquine group while it was 1.5% in the pioglitazone group when used as add-on to metformin and sulfonylurea [12].

While GI side-effects were more frequent among hydroxychloroquine treated patients. This AE was at comparatively high with maximum dose of hydroxychloroquine i.e. with 400 mg. For this reason further it was studied that if hydroxychloroquine was given at divided dose of 200 mg BD instead of 400 mg OD what could be the difference in efficacy and GI tolerance. It has been seen that when hydroxychloroquine 400 mg was substitute with 200 mg BD there was almost same efficacy in reducing further glycemic parameters and number of patients who earlier complain of GI disturbance and other GI related side effect were significantly decreased.

The anti-inflammatory drugs can be important in the prevention of major cardiovascular event like MI, stroke, and cardiovascular death. Some evidence is available for the beneficial impact of hydroxychloroquine on cardiovascular risk, particularly in diabetes and dyslipidemia. The anti-inflammatory agents frequently prescribed in RA or psoriatic arthritis are being evaluated in the management of CVD. Similarly, the role of hydroxychloroquine has been already established to control CVD like MI in high risk individuals such as RA patients.

Cardiac safety studies, particularly the electrophysiological implications for HCQ, have predominantly been evaluated on the basis of its use as an antimalarial treatment and prophylactic. When looking to repurpose hydroxychloroquine for managing diabetes patients, it is essential that the presence or absence of QT interval prolongation in patients are taken into consideration [31]. A retrospective study (from January 1, 2001–October 31, 2013, excluding patients with CVD prior to RA diagnosis) done by Sharma TS et al., confirms that hydroxychloroquine use was associated with a 72% decrease in the risk of incident CVD in RA patients [32]. The authors also commented that the biological plausibility of this protective association is supported by the favourable associations of hydroxychloroquine with glucose and lipids in RA patients and with thrombosis in lupus patients and nonrheumatic patients [32]. A retrospective study recently published has also confirmed the cardiac safety of hydroxychloroquine [33]. In this study researcher evaluated effect of hydroxychloroquine/Chloroquine (HCQ/CQ) on TdP/QT using data from the U.S. Food and Drug Administration’s Adverse Event Reporting System (FAERS) (> 13 million total report analysed incorporating extensive safety data of last 50 rears, 1969–2019). Lower 95% Cls for HCQ/CQ alone showed no potential safety signals for TdP/QT prolongation, death or accident/injuries or depression [PRRs and 95% Cls for TdP/QT prolongation was 1.43 (1.29–2.59)] [33]. As a cardio vascular safety hydroxychloroquine also has been associated with beneficial changes in lipid profiles, including decrease in low- density lipoprotein and total cholesterol, which result in a less atherogenic lipid profile [34–36]. Even prior to the advances in current antithrombotic therapies, hydroxychloroquine was used perioperatively for deep venous thrombosis prophylaxis in abdominal and orthopaedic surgeries [37–39]. Studies had already confirmed that hydroxychloroquine use was associated with reduced risk of thrombotic events in patients with lupus and antiphospholipid syndrome [40]. Even it had been found that hydroxychloroquine acts as a bradycardic agent in SAN cells, in atrial preparations and in vivo and lows the rate of spontaneous action potential firing in the SAN through multichannel inhibition, including that of I_f_ [41]. This documentations probably explains the protective CVD association seen with hydroxychloroquine which can be even true in patients with diabetes.

Ocular toxicity is the most important concern linked with chronic use of hydroxychloroquine. To manage ocular risk, use of minimum effective dose of hydroxychloroquine and periodic ophthalmological screening is recommended. We have already used recommended dose of hydroxychloroquine in this current trial as per recommendation from AAO to avoid ocular toxicity. A recent findings from large cohort study (*N* = 2867) from Cleveland with follow-up from 1999 to 2017 has confirm that only 31 patients had visual impairment and majority were due to comorbidities such as diabetes, hypertension, stroke, cardiac arrest etc. [42] This study also confirmed that even after long-term use in diabetic patients HCQ is safe [42]. It recommended that, the baseline ophthalmological screening should be done before initiation of hydroxychloroquine therapy and yearly screening should be performed after five years of use when risk factors are absent. Regular yearly ophthalmological screening is recommended for the patients with risk factors.

Like many other countries in the world India has also been affected by COVID-19 pandemic and at the time of writing this article, there are already more than 100,000 cases of COVID-19 infection, and more than 3000 individuals have died of the disease in India. Emerging evidence suggest that for COVID-19, most prevalent comorbidities id diabetes and also for a worse prognosis of the disease it is one of the major risk factors [43–51]. Antihypoglycemic property of hydroxychloroquine in patients with diabetes and the risk benefits at the time of COVID-19 pandemic is major subject of discussion. Emerging evidence with regards to the current pandemic scenario shows that following COVID-19 infection diabetic patients have a greater risk for adverse outcomes [52]. There are several review articles published which support the effect of hydroxychloroquine in the early infection stage of COVID-19 [53–55]. Further more recent study confirms that countries who were using antimalarial drugs has much lesser death rates as compare to countries which do not [56]. Although findings from clinical studies have also suggested limited benefit from hydroxychloroquine in COVID-19 in general [57], several randomized controlled trials are currently investigating the use of HCQ for prophylaxis of COVID-19 [58]. Moreover, guidelines from different countries have listed some investigational drugs (including hydroxychloroquine) as potential adjuvant treatment options [57, 59].

Thus the authors of this trial hope that the findings from this RCT will help great number of uncontrolled diabetes patients in developing country like India to achieve their glycemic target.

Sample size of the trial was small, further large scale data with longer duration of time thorough ophthalmologic monitoring and cardiovascular outcomes assessments required to established further effectiveness of different doses of HCQ as add-on treatment for T2DM.

## Conclusion

Hydroxychloroquine added to sulphonylurea and metformin, improves glycemic control significantly in T2DM patients. Glycemic effect of different dose of hydroxychloroquine is dose dependent. The safety/tolerability profile of hydroxychloroquine was favourable except GI disturbance which more frequent with 400 mg. This can be avoided with 200 mg BD without compromise on efficacy. Even type 2 diabetes patients who were below 60 kg of body weight can get the benefit from lower dose of hydroxychloroquine i.e., hydroxychloroquine 200 mg and hydroxychloroquine 300 mg. We have shown that weight based dosing can be an ideal option for choosing hydroxychloroquine as an add-on antidiabetic treatment.

## Abbreviations

T2DM: Type 2 diabetes mellitus management
HbA1c: Glycated haemoglobin
FPG: Fasting plasma glucose
PPG: Post prandial plasma glucose
HCQ: Hydroxychloroquine
